# The synthesis and evaluation of thiolated alginate as the barrier to block nutrient absorption on small intestine for body‐weight control

**DOI:** 10.1002/btm2.10382

**Published:** 2022-08-12

**Authors:** Tzu‐Chien Chen, Rui‐Chian Tang, Jhih‐Ni Lin, Wei‐Ting Kuo, I‐Hsuan Yang, Ya‐Jyun Liang, Feng‐Huei Lin

**Affiliations:** ^1^ Department of Biomedical Engineering, College of Medicine and College of Engineering National Taiwan University Taipei Taiwan; ^2^ Institute of Biomedical Engineering and Nanomedicine, National Health Research Institutes Zhunan, Miaoli County Taiwan

**Keywords:** alginate, barrier, health supplement, obesity, thiol group

## Abstract

Obesity is the most common health concern all over the world. However, till now, there is no promising way to manage obesity or body‐weight control. The aim of the study is to develop an edible gel as a health supplement that temporarily attaches to the mucus of the intestines, forming an absorption barrier to block the nutrients. We modify the alginate with the thiol group as thiolated alginate (TA) that may stay on the mucosa layer for a much longer time to reduce nutrient absorption. In this study, the TA is synthesized successfully and proved a good mucosal adhesion to serve as a barrier for nutrient absorption both in vitro and in vivo. The results of in vivo imaging system (IVIS) show that the synthesized TA can be exiled from the gastrointestinal tract within 24 h. The animal study shows that the TA by daily oral administration can effectively reduce body weight and fat deposition. The biosafety is evaluated in vitro at the cellular level, based on ISO‐10993, and further checked by animal study. We do believe that the TA could have a greater potential to be developed into a safe health supplement to manage obesity and for body‐weight control.

## 1 INTRODUCTION

Overweight or obesity is defined as abnormal or excessive fat accumulation that would be a risk to health. A body mass index (BMI) over 25 is considered overweight, and over 30 is obese.^1^ Obese individuals are at risk for a wide range of health problems, including high blood pressure, coronary artery disease, cerebrovascular disease, atherosclerosis, renal insufficiency, and particularly diabetes, which currently affects 422 million adults worldwide.^2^, ^3^ Accordingly, the World Health Organization (WHO) estimates that there are 2.3 billion overweight adults worldwide and 700 million obese adults.^4^, ^5^ The issue has grown to epidemic proportions, with over 4 million people dying each year as a result of being overweight or obese in 2017 according to the global burden of disease.^6^

The etiology of obesity is considered to be an energy imbalance resulting from excessive calorie intake or insufficient calorie expenditure.^7^ Therefore, limiting intestinal absorption might be an effective strategy. Bariatric surgery such as Roux‐en‐Y gastric bypass, sleeve gastrectomy, and laparoscopic adjustable gastric banding has been demonstrated by prior research to be one of the most effective ways to help obese patients lose weight.^8^ Although the demand for bariatric surgeries is increasing, alternatives to the obese and diabetics have been developed to combat the growing epidemic of obesity and diabetes. As a less invasive approach, EndoBarrier is an endoscopically applied device for obesity and type II diabetes.^9^ Being a hollow soft plastic tube with a metal ring on the top, EndoBarrier is fixed to the pyloric sphincter via gastroscopy.^10^ The small intestine is the main absorption site of nutrients, and the EndoBarrier blocks nutrient absorption from the duodenum through the polymer film. Consequently, EndoBarrier improves the comorbidities related to obesity, such as nonalcoholic fatty liver disease, type 2 diabetes, and dyslipidemia.^11^ However, users may experience stomach pain and nausea; more, the intestinal peristalsis may cause EndoBarrier to fall off, and the device can stimulate abnormal proliferation by pushing surrounding tissues and needed to be removed within 1 year.^12^ Therefore, referring to the essential aspects of Endobarrier, our goal is to develop an edible gel as a health supplement that temporarily attaches to the mucus of the intestines, forming an absorption barrier to block the nutrients. The gel would gradually detach from the small intestinal tract within 24 h.

Alginate, a seaweed compound, is a biocompatible, biodegradable, and nontoxic natural polymer. It is widely used in drug and cell delivery systems.^13^ Evidence has shown that alginate inhibits digestive enzymes like α‐amylase, α‐glucosidase, pepsin, and lipase.^14^, ^15^ Thus, alginate has been implicated as a potential agent for obesity treatments. However, many studies revealed that the results of daily alginate administration were not so promising for body‐weight control.^16^ That may be owing to the alginate being water‐soluble, which may cause fast gastrointestinal passage. Therefore, we try to extend the retention time of alginate and shortly cover the alginate on the mucous layer of the gastrointestinal tract to reduce the nutrient intake to achieve the goal of anti‐obesity.

In the microenvironment of the small intestine, the mucus layer of the small intestine contains cysteine on certain glycoprotein domains, such as muc2, a major component of the mucus layer secreted by goblet cells.^17^ In addition, the disulfide bonds between glutathione/glutathione and cysteine/cystine would be formed and broken down to keep redox reactions happening in the small intestinal tract.^18^ Based on the mechanism, if we could modify the alginate with the thiol group to join the disulfide bonds formation and breaking‐down with glutathione and cysteine in the small intestinal tract, we could expect that the TA may stay on the mucosa layer for a much longer time. Furthermore, the studies told that disulfide bonds formed by thiomer and mucin were reversible,^19^ indicating that the thiol‐modified alginate could short‐term adhere to the small intestinal tract by disulfide bonds formation and then detach from the mucosa layer by disulfide bonds breaking‐down to prevent from accumulation.

In the study, the synthesized TA was characterized by functional groups identification, molecular structure analysis, and elements measurement, respectively. Furthermore, the mucoadhesive and barrier properties were evaluated in vitro, along with cell viability and cytotoxicity assays. As for animal studies, C57BL/6 male mice were used for the in vivo imaging system (IVIS) and the functional barrier test. Finally, a high‐fat diet (HFD) mice model was utilized to evaluate the positive effects of TA on body‐weight gain and adipose tissue accumulation.

## 2 RESULTS AND DISCUSSIONS

### 2.1 Material characterization of synthesized TA

**FIGURE 1:**
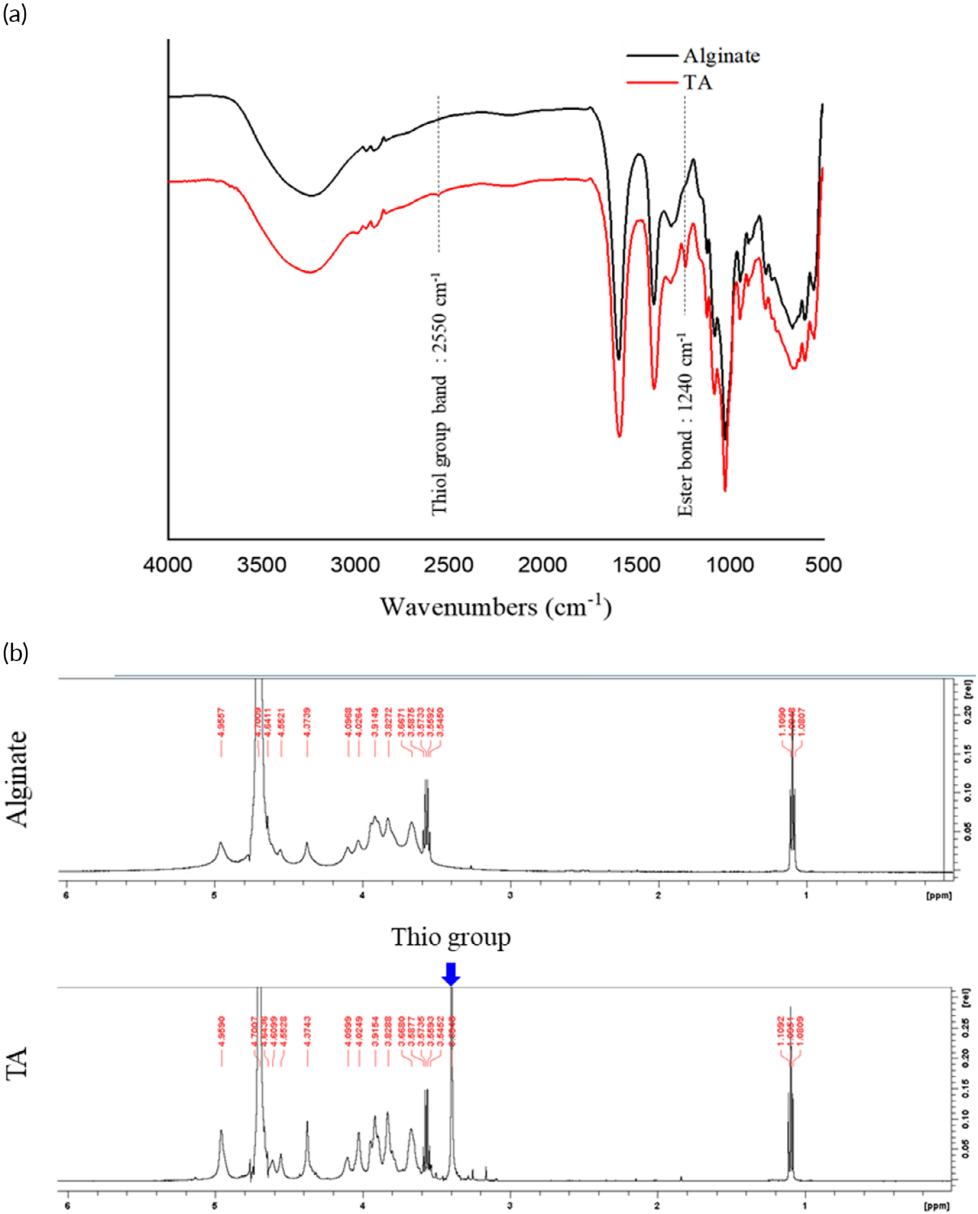
Fourier transform infrared (FTIR) and ^1^H NMR spectra of alginate and thiolated alginate (TA). (a) The pattern by black and red were the FTIR spectrum for alginate and TA, respectively. The absorption bands at 2500 and 1240 cm^−1^ were corresponding to the thiol group and ester bond of the synthesized TA, respectively. (b) Upper pattern and lower pattern were the ^1^H NMR spectra for alginate and TA, respectively, where the chemical shift at 3.4 ppm as indicated in the blue arrow was assigned to the thiol group on TA.

Fourier transform infrared (FTIR) was used to analyze the functional group of the synthesized TA. As shown in Figure 1a, the absorption band at 1240 and 2550 cm^−1^ where was identified as COC— ester bond and thiol group of the synthesized TA, respectively. The results showed that the thioglycolic acid (TGA) was successfully grafted to the alginate through the COOH— and OH— linkage to left a thiol tail group for di‐sulfur bond formation in the GI tract.

The molecular structure of TA was characterized by ^1^H NMR. As shown in Figure 1b, the chemical shift at 3.4 ppm was corresponding to the thiol group. The result was to further approve that the TGA was grafted to the alginate molecules. The calculation of the degree of substitution of TA: The characteristic peak at 4.70 ppm represents the anomeric proton of M and H‐5 of G‐units adjacent to M, while the characteristic peak at 3.40 ppm represents the CH_2_ of the TGA. Due to the number of protons, the integral area ratio of the two peaks would be 1:2. Therefore, the degree of substitution may be calculated by (real peak area ratio)/(1:2). For thiolated alginate, the degree of substitution would be 0.01, equivalent to 1%. On the other hand, the sulfur signal could also be traced on the EDS spectrum, which further confirmed the successful conjugation of thioglycolate, as shown in Figure S1. Interestingly, the two experimental results of EDS and ^1^H NMR were consistent.

### 2.2 Biocompatibility of the synthesized TA

**FIGURE 2:**
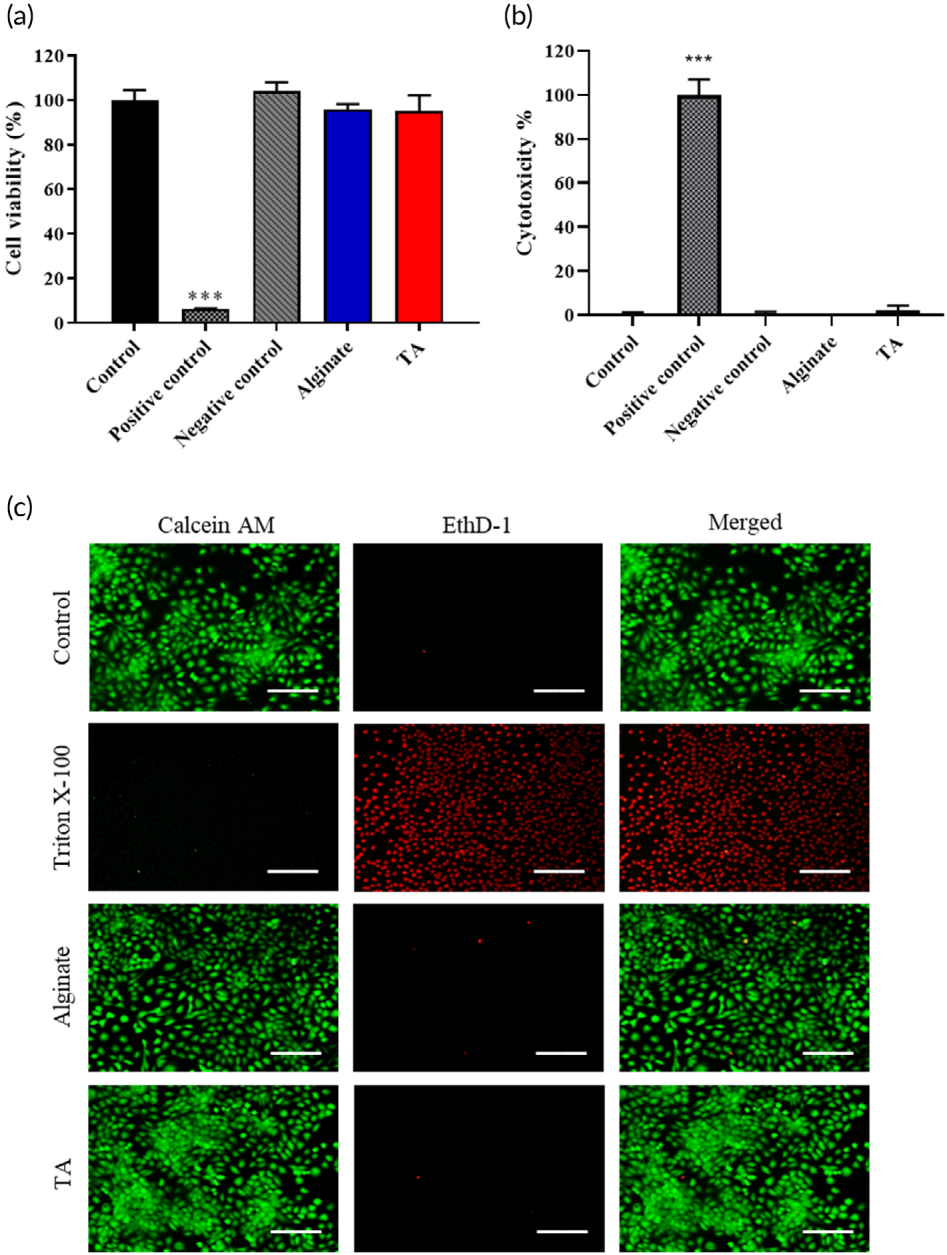
The biocompatibility of alginate and thiolated alginate (TA) by water‐soluble tetrazolium salt‐1 (WST‐1), lactate dehydrogenase (LDH) assay, and live/dead staining. (a) The evaluation of cell viability of alginate and TA by WST‐1 assay; (b) the evaluation of cytotoxicity of alginate and TA by LDH assay; and (c) the cell death rate of alginate and TA by live/dead staining, in which the living cells were in green and the dead cells were shown in red (scale bar = 100 μm).

The result of cell viability to synthesized TA was about 94.5%, identified as a nontoxic level (Figure 2a). The result of LDH was around 1.56%, which was as low as the baseline (control group) (Figure 2b). Figure 2c was the live/dead staining to be in terms of cell death rate; where the red color represented the dead cells and the green color represented the living cells. The results were quantified by the ImageJ software. The cell death rate was as low as the control group, which was treated with culture medium. From the results of WST‐1, LDH, and live/dead staining, we could tell that the synthesized TA would be proved in “no toxic level” to L929 cells.

### 2.3 Mucoadhesive test of TA in vitro

**FIGURE 3:**
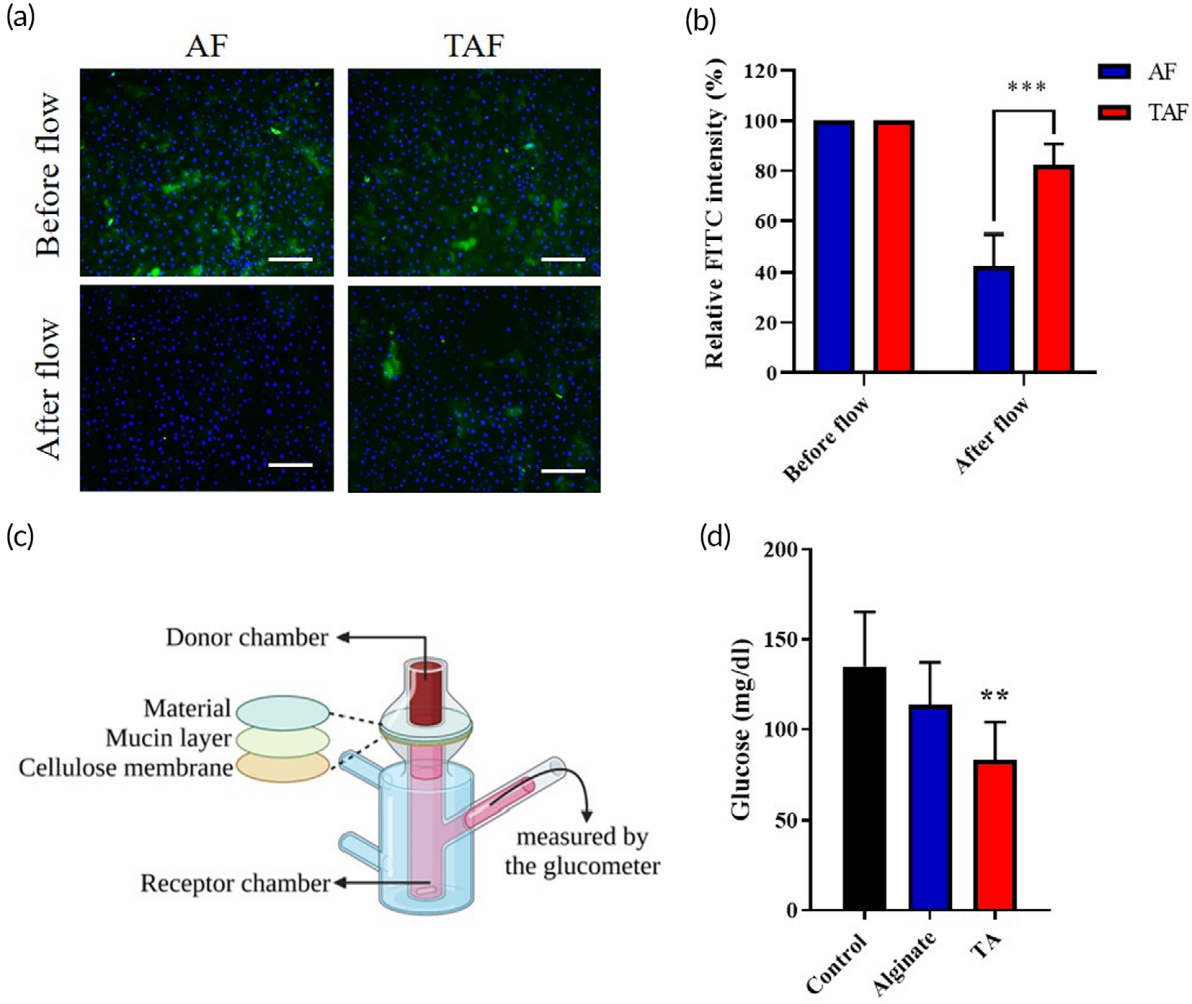
The in vitro assessment of adhesion ability and the evaluation of barrier function of TA by μ‐Slide and modified Franz‐type diffusion cell, respectively. (a) The IEC‐6 cells were cultured on μ‐Slide and then coated with alginate‐FITC (AF) and TA‐FITC (TAF); followed to wash with culture medium by constant flow stress. The blue is the contrast stain of the nucleus and the green is the FITC fluorescence, which would be in terms of the retention rate of AF and TAF. (scale bar = 100 μm); (b) after washing with medium, the μ‐Slide would be observed under a fluorescent microscope; on which the relative intensity of FITC was quantitative by ImageJ software to know how much AF or TAF was left on μ‐Slide to evaluate the adhesion ability of synthesized TA in vitro. One‐way ANOVA (*n* = 3, ***p < 0.001); (c) the schematic diagram of the barrier function test in a modified Franz‐type diffusion cell; (d) the results of the investigation of TA as a nutrient barrier in the small intestine by a modified Franz‐type diffusion cell. One‐way ANOVA with multiple comparisons (*n* = 6 for each group, **p < 0.01 compared to control)

An in vitro model of μ‐slides was designed to evaluate the mucoadhesive ability of synthesized TA on the small intestinal epithelium. The intensity of green fluorescence would indicate how much of alginate‐FITC (AF) or thiolated alginate‐FITC (TAF) is bound to the mucous layer through a disulfide bond. As seen in Figure 3a, the blue fluorescence on the AF group and TAF group were not significantly different at 2 h after constant medium flow over the μ–Slides. The result indicated that the seeded cells would not be detached from the μ–Slides under applied flow rate. The green fluorescence at 0 h was normalized to 100% to serve as the ceiling level. At the second hour, the retained TAF on the μ–Slides was approximately 82.28%, which was much higher than the retained AF of 42.38% (Figure 3b). The result might tell that TAF should have a better adhesive property than that of AF. We could expect that TAF might have a higher covering time on the small intestine to block the nutrient absorption.

### 2.4 The investigation of TA as a nutrient barrier in vitro

A modified Franz‐type diffusion cell was used in this study as the in vitro model to evaluate whether TA could serve as a nutrient barrier to inhibit glucose absorption (Figure S2). The cellulose membrane was coated with mucin to simulate the mucus layer of the intestinal tract, followed by adding TA to evaluate its ability to inhibit glucose penetration. After free diffusion for 10 min, the diffused glucose was collected from the receptor chamber (ground part) of the modified Franz‐type diffusion cell and then measured by the glucometer Figure 3c. The glucose concentration of the control group was 134 mg/dl as shown in Figure 3d. The glucose concentration of the TA group was about 83 mg/dl, which was 38.06% lower than that of the control group. On the contrary, the glucose concentration of the alginate group was 113 mg/dl, which was much higher than that of the TA group. Therefore, we believe that TA could effectively bind to the mucin layer to block glucose permeation.

### 2.5 The mucoadhesive test of TA in vivo

**FIGURE 4:**
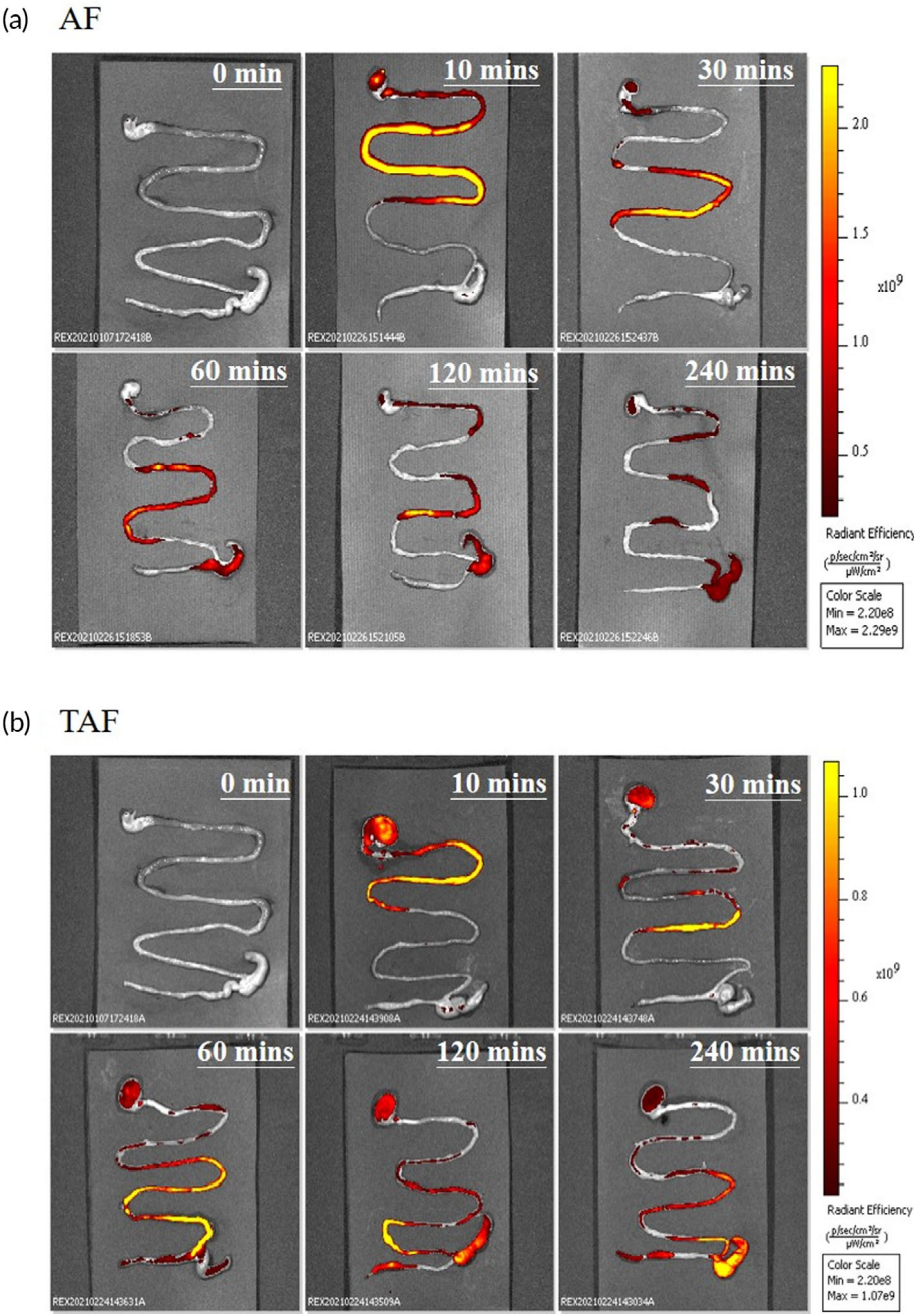
Evaluation of TA retention on the GI tract in vivo. Mice were gavaged with (a) AF of 100 mg/kg and (b) TAF of 100 mg/kg. The gastrointestinal tracts were harvested from the stomach to colon at each time point and analyzed with IVIS in vivo that would be imaged by a pseudo‐color to show the intensity reduced from yellow to red.

C57BL/6 male mice were used to verify the adhesion property of AF or TAF on the small intestinal mucosa. At the first 10 min, there was no significant difference for the fluorescent distribution between the AF group and the TAF group as shown in Figure 4a. After 60 min, the fluorescence of the TAF group could be observed from the stomach, duodenum to the colon (Figure 4b). However, the AF group showed a high GI passing rate; with which the fluorescence could only be observed in the lower part of the GI tract (Figure 4a).

At 240 min, the fluorescence of the AF group could be observed only in several parts of the GI tract sporadically (Figure 4a). Nevertheless, the fluorescence in the TAF group covered the whole GI tract examined from the stomach, small intestine to the colon. Based on the results, TA could remain on the GI tract for a longer period with a lower GI passing rate. The fluorescence of the TAF group was all cleared from the GI tract (Figure S3), indicating that it could achieve temporary barrier effects to block the nutrient absorption, rather than staying on the GI tract for a long time that may interfere with food digestion.

### 2.6 The functional barrier test by OGTT and IPGTT

**FIGURE 5:**
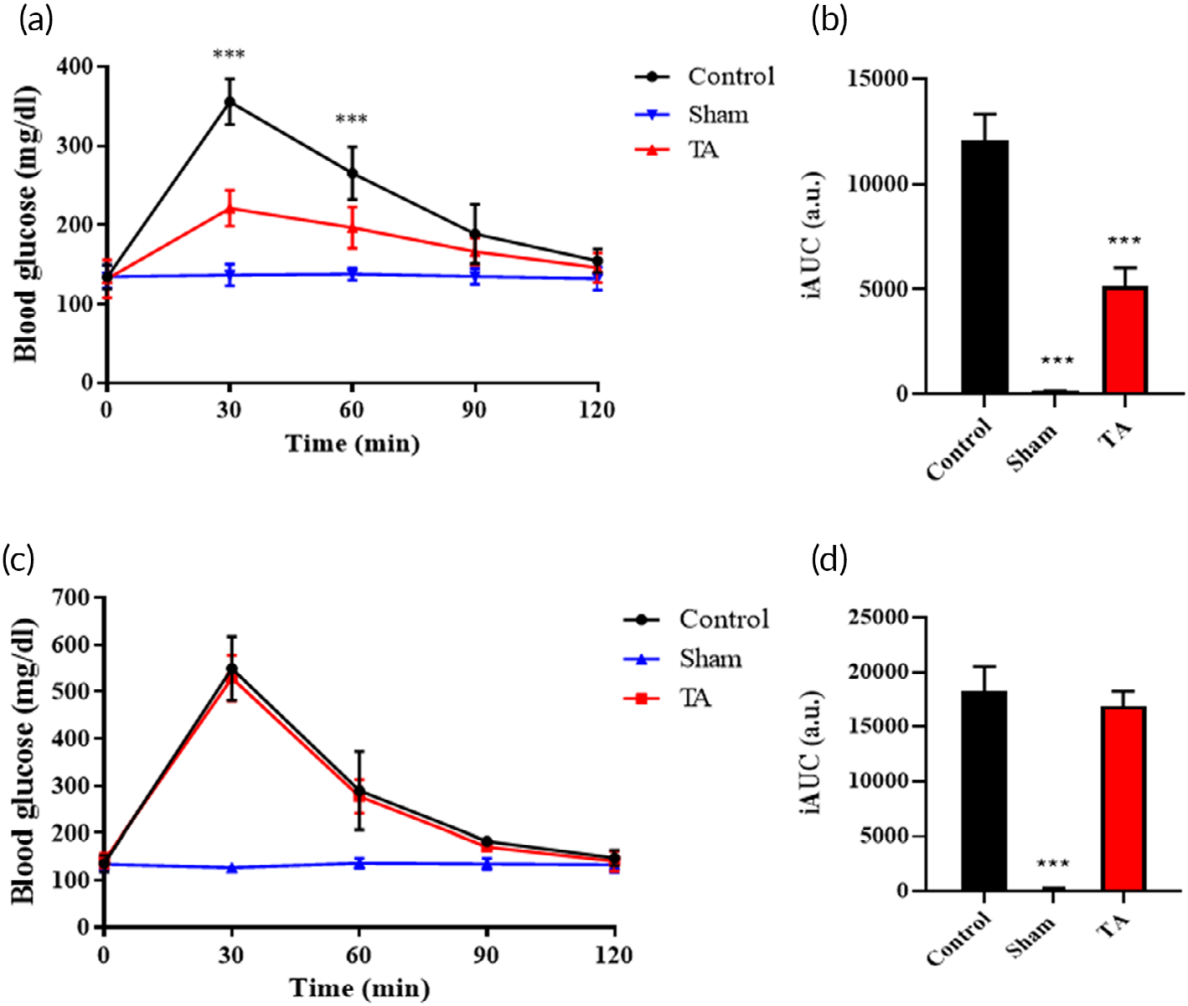
Evaluation of mucus adhesion in vivo and thiolated alginate (TA) administration with reduced glucose response. (a) Oral glucose tolerance test (OGTT) curves in black, red, and blue were mice gavaged by PBS followed with glucose solution, TA followed with glucose solution, and PBS only; that was indicated as the control group, TA group, and sham group, respectively. One‐way ANOVA with multiple comparisons (*n* = 10, ***p < 0.001); (b) the incremental area under curve (iAUC) of Figure 5a. One‐way ANOVA with multiple comparisons (*n* = 10, ***p < 0.001 compared to control); (c) intraperitoneal glucose tolerance test (IPGTT) curves in black, red, and blue were the mice oral administrated with PBS and TA and then followed with glucose injection to peritoneal cavity indicated as the control group and TA group, respectively; whereas the mice with PBS oral administration without glucose injection was assigned as the sham group. One‐way ANOVA with multiple comparisons (*n* = 6, ***p < 0.001); (d) the iAUC of the IPGTT curves. One‐way ANOVA with multiple comparisons (*n* = 6)

To assess the barrier function in vivo of TA on nutrient absorption, oral glucose tolerance test (OGTT) was used to check whether the TA could bind to the mucous layer of the small intestine to block glucose absorption. As shown in Figure 5a, the glucose level in the blood of negative control was kept within a normal level. At 30 min, the blood glucose level of the TA group was about 221 mg/dl, which was much lower than that of the control group (355 mg/dl). At 120 min, the glucose concentration in the blood all returned to baseline level no matter in the TA group or the control. Figure 5b was the incremental area under the curve (iAUC) of Figure 5a.

The intraperitoneal glucose tolerance test (IPGTT) was to further confirm that the gavaged TA would only block glucose absorption by the GI tracts rather than other pathways. As shown in Figure 5c, there was no significant difference. The results showcased that the curve was fully matched in the control and TA group. The AUC also showed no significant difference in the two groups (Figure 5d). From the results of OGTT and IPGTT, we believe that TA could bind to the mucous layer of the small intestine as a barrier to glucose absorption.

### 2.7 The efficacy of TA on body‐weight control and adipose tissue accumulation in the animal study

**FIGURE 6:**
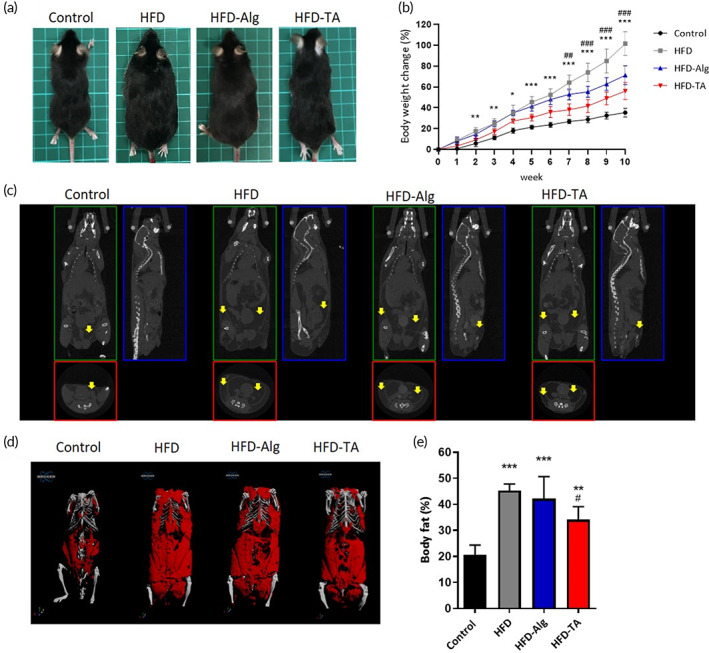
TA reduces weight gain, CT imaging, and glucose response. (a) The photographs were the gross examination of the mice appearance from the control, HFD, HFD‐Alg, and HFD‐TA at the end of the experiment (scale bar = 1 cm); (b) the curves in black, gray, blue, and red were the body‐weight gain versus experimental period of the mice in Control, HFD, HFD‐Alg, and HFD‐TA, respectively. One‐way ANOVA (*n* = 6, *p < 0.05, **p < 0.01, ***p < 0.001 for HFD‐TA compared with HFD; ##*p* < 0.01, ###*p* < 0.001 for HFD‐Alg compared with HFD); (c) the CT scan of the mice from control, HFD, HFD‐Alg, and HFD‐TA was imaged with a resolution voxel spacing of 35 μm. The white adipose tissue was indicated as a light‐yellow arrow; (d) the whole‐body fat distribution from the mice were recorded in red color, respectively; and the white color was the skeleton of mice; (e) the quantitative data of whole‐body fat from the previous images. One‐way ANOVA multiple comparisons (*n* = 6, **p < 0.01,***p < 0.001 compared with control group; #*p* < 0.05 compared with HFD group)

For the long‐term evaluation, a high‐fat diet (HFD) induced C57BL/6 male mice were used to evaluate the efficacy of TA on body‐weight control. At the end of the experiment, the gross examination of mice was as shown in Figure 6a, in which the mice size in the HFD group (high‐fat diet with PBS gavaged) was the biggest one, much bigger than that of the control group (normal diet with PBS gavaged), HFD‐Alg group (high‐fat diet with alginate gavaged) and HFD‐TA group (high‐fat diet with TA gavaged). Figure 6b was the body weight recorded every week of the three groups; among those, the body weight of the HFD group showed the highest increasing rate. Although the body‐weight gain of the TA group was not as low as the control group, it was much lower than the HFD group and the HFD‐Alg group. Specifically, the TA group had a 44.85% body weight decrease at the end of the 10‐week‐experiment and also had a significant difference in only 2 weeks compared with the HFD group.

CT scan was utilized to evaluate the visualized body fat accumulation (Figure 6c). Figure 6d was the image of 3D adipose tissue rendered from Figure 6c by equipped software of the CT‐scan, in which the red color was the adipose tissue or fat tissue. We could see that the fat tissue was thickly covered on the whole body in the HFD group, whereas the HFD‐TA group was not so severe in fat tissue accumulation. Figure 6e was the quantitative results calculated from Figure 6d. Although the adipose tissue accumulation of the HFD‐TA group (35.85%) was not as less as the control group (20.59%), it still showed much lower than that of the HFD group (45.83%) and the HFD‐Alg group (42.23%) significantly.

### 2.8 The efficacy of TA on adipose tissue formation

**FIGURE 7:**
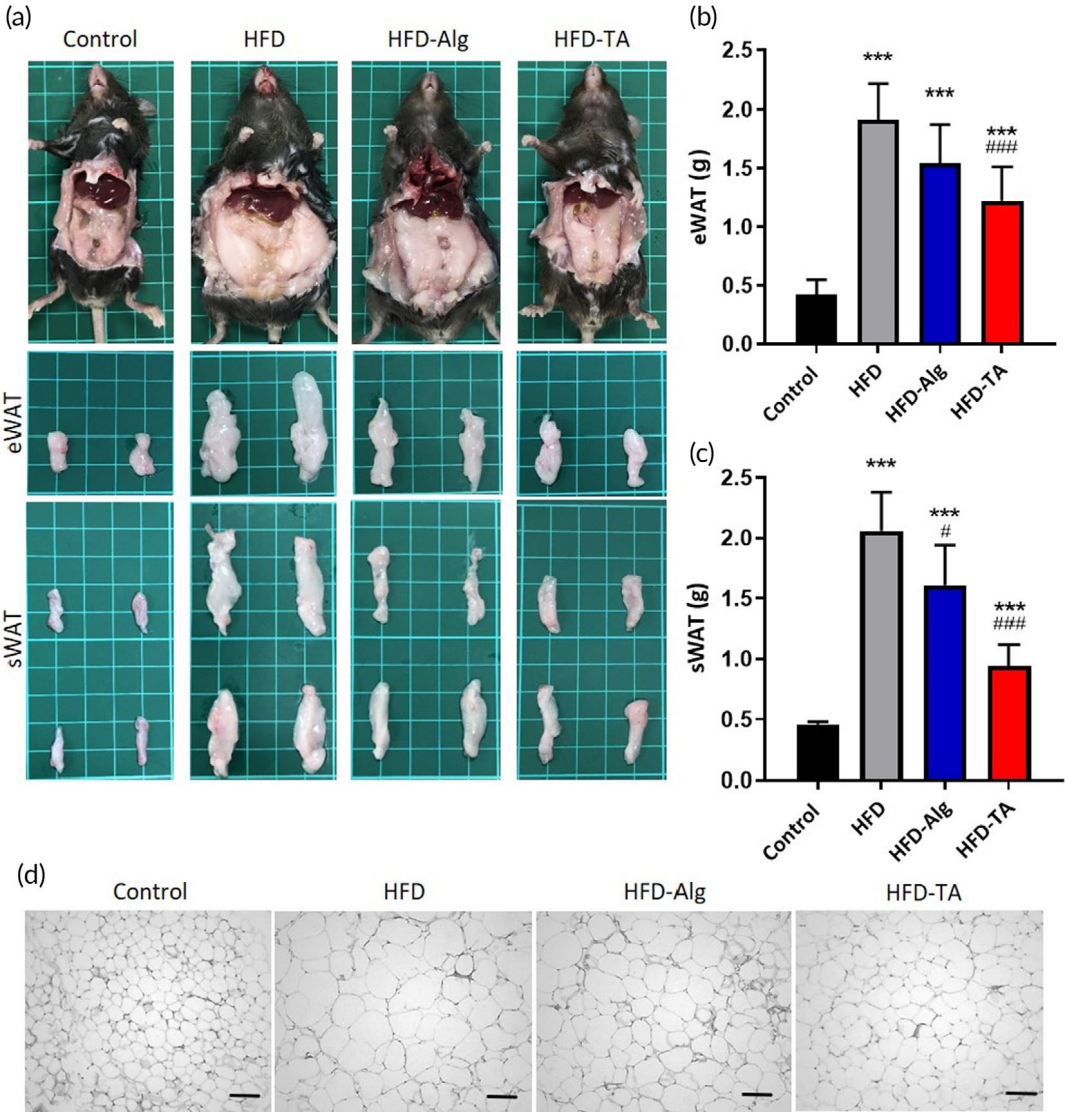
Therapeutic effects of TA on body fat deposition in HFD‐induced mice model. (a) the mice from control, HFD, HFD‐Alg, and HFD‐TA were sacrificed and then observed the epididymal white adipose tissue (eWAT) and abdominal subcutaneous white adipose tissue (sWAT); (b) the weight of eWAT. One‐way ANOVA with multiple comparisons (*n* = 6 for each group, ***p < 0.001 compared with control group; ###*p* < 0.001 compared with HFD group); (c) the weight of the sWAT. One‐way ANOVA with multiple comparisons (*n* = 6 for each group, ***p < 0.001 compared with control group; #*p* < 0.05, ###*p* < 0.001 compared with HFD group); (d) H&E staining of eWAT (scale bar = 100 μm)

The animals were sacrificed in the 10th week of the study. Epididymal white adipose tissue (eWAT) and subcutaneous white adipose tissue (sWAT) were harvested as shown in Figure 7a. By gross examination, both eWAT and sWAT were significantly reduced in the HFD‐TA group compared to that of the HFD group and the HFD‐Alg group. The quantitative results of eWAT by weight were shown in Figure 7b, where the eWAT in the HFD‐TA group was 36.05% lower than that of the HFD group and 19.38% lower than that of the HFD‐Alg group. The sWAT of the HFD‐TA group was also lower than that of the HFD group by 54.37% and that of the HFD‐Alg group by 21.84%, as shown in Figure 7c. As shown in Figure 7d, the size of adipocyte of eWAT in the HFD group was much larger than in any other group.

## 3 DISCUSSIONS

The gastrointestinal mucosal barrier is made up of epithelial and immune cells, which together form a barrier to blockade harmful substances.^20^ The epithelial cells are covered by a thick layer of mucus, which serves as the first line of innate immune defense. Mucus forms a protective barrier that prevents microorganisms and noxious substances to penetrate the surface of the epithelium. The major building blocks of the mucus gel are high‐molecular‐weight glycoproteins called mucins.^21^ Mucins belong to two distinct families: secreted mucins and transmembrane mucins. They are subdivided into MUC1 to MUC213 according to their functions.^22^ The secreted mucins are responsible for the formation of the mucus layer over the epithelium. The mucins contained a lot of cysteine (Cys) and glutathione (GSH). Cys and cystine (CySS) are the predominant thiol/disulfide pools found in the plasma and are essential to maintain the redox state of plasma proteins during the digestion of food.^23^ Cys is also involved in protein and glutathione (GSH) synthesis, as well as taurine and sulfate synthesis. The detoxification enzymes of GSH and sulfate perform diverse functions such as transporting bile salts and osmotic regulation.^24^

As shown in previous studies, we could summarize that the coupling of Cys/CySS and GSH/GSSG systems play a crucial role in intracellular redox balance and antioxidant function in the GI tract during the food digested, especially in the small intestine. As known, the small intestine is the most important area where the absorptive function occurs. Therefore, if we could limit intestinal absorption, that would be an effective strategy to fight obesity or for body‐weight control. In this study, TA was synthesized by TGA grafted onto the alginate. The hydroxyl groups on alginate were bound to the carboxyl groups on TGA by ester linkage and provided thiol groups to react with the free thiols (Cys and GHS) of mucin covering the small intestine. Through the formation of di‐sulfur bonds between TGA and free thiols on mucin, the TGA may temporarily cover the mucous layer of the small intestine to be a barrier to the absorption of nutrients to achieve body weight control.

Alginate, a water‐soluble biopolymer, has been widely applied in the food industry, pharmaceutical and biomedical medicine industry as delivery systems.^25^ It has been developed into food or health supplements against obesity.^26^ Although it could provide additional satiety in the stomach to inhibit eating desiring, the results to manage obesity were not so convincing. As mentioned, alginate is a water‐soluble biopolymer, which could not stay in the GI tract for a much longer time. In the study, alginate was therefore used as the matrix material grafted with thioglycolic acid, abbreviated as TGA, which may bind to mucous layer by di‐sulfate bonds and stay in the GI tract for a longer time. The TA could temporarily cover the mucous layer through mucin reversible redox reaction, to serve as a barrier to reduce the food intake.

TA has been synthesized to prove by FTIR and NMR. The binding ability of TA to mucin was checked in vitro and in vivo by μ‐Slide and IVIS, respectively. The function to serve as a barrier for nutrient absorption of TA was confirmed by a modified Franz diffusion cell in vitro. Also, OGTT and IPGTT successfully confirmed the barrier function in vivo. Moreover, the IEC‐6 cell line also underwent the WST‐1, LDH assay, and live/dead staining shown in Figures S4–S6. The results were similar to Figure 2, we confirmed that TA is biocompatible to the IEC‐6 cell line, indicating the biosafety of the specimen on the small intestinal epithelial cells. As shown in Figures 3 and 4, TA had better adhesion properties than the unmodified alginate in the results of the μ‐Slides test and IVIS. Even though TAF showed better attachment ability than AF, the results indicated that TAF was more likely to remain in the lower part of the GI tract, whereas AF was more likely to attach to the upper part of the GI. The interaction of the material and the small intestine might be separated into different types of small intestines such as duodenum jejunum and ileum. It is worth exploring in future studies detailly.

Furthermore, TA also inhibited glucose absorption by forming a coating on the small intestine as a nutrient barrier in Figure 5. Moreover, via the formation of di‐sulfur bonds between TA and small intestine, TA may temporarily cover the mucous layer of the small intestine to inhibit the absorption of nutrients to achieve body‐weight control in Figures 6 and 7. However, the food intake shown in Figure S7, implied that the reason for body‐weight control is not to inhibit food intake but rather to inhibit absorption. At the end of the animal experiment after 10 weeks, the H&E staining of the stomach, small intestine, pancreas, and spleen did not show significant lesions (Figure S8). Consequently, these results once again showed that it is a biocompatible material in the animal study.

In our study, TA forms a transient coating over the intestinal tract, which mimics the ability of EndoBarrier in a noninvasive manner. TA not only eliminates the risk of abnormal tissue proliferation and detachment of the EndoBarrier but may also greatly improve patient compliance. However, most current research on the gastric barrier is based on obesity. As such, future studies may focus on assessing the efficacy of TA treatment after establishing human obesity models, in order to acquire a more comprehensive understanding of the efficacy of TA versus existing solutions.

## 4 CONCLUSION

In this study, the TA was successfully synthesized by grafting thiols onto the alginate. TA has been proved a good mucosal adhesion by di‐sulfate bond formation; that could serve as a barrier for nutrient absorption both in vitro and in vivo. From the results of IVIS, the synthesized TA could be exiled from the GI tract within 24 h. The animal study showed that the TA by daily oral administration would effectively reduce the body weight and fat deposition. The biosafety was evaluated in vitro at the cellular level and further checked by animal study. We do believe that the TA could have a greater potential to be developed into a safe health supplement for body‐weight control.

## 5 MATERIALS AND METHODS

### 5.1 Reagents

Sodium alginate, thioglycolic acid (TGA), sodium bicarbonate, 1‐ethyl‐3‐(3‐dimethylaminopropyl) carbodiimide (EDC), *N*‐hydroxysuccinimide (NHS), 1,6‐diaminohexane, ethanol were obtained from Sigma‐Aldrich. Dulbecco's Modified Eagle's Medium (DMEM) and mini‐mum essential medium (MEM), fetal bovine serum (FBS), and antibiotic‐antimycotic were as received from Sigma‐Aldrich, Hyclone, and Gibco, respectively. Insulin transferrin selenium mixture (ITS‐M) was purchased from GeneDireX. WST‐1, LDH, and live/dead stain assay were obtained from Roche, Takara, and Thermo Fisher, respectively. Trypsin–EDTA, calcein AM, ethidium homodimer‐1 (EthD‐1), propidium iodide, Hoechst 33342 were bought from Thermo Fisher as well.

### 5.2 The preparation of TA

The preparation of TA was to conjugate the TGA to alginate molecules by the dehydration of the COOH− group and OH− group, respectively, for the formation of the ester linkage. A 8 g of sodium alginate was dissolved in 120 ml of ddH_2_O and then mixed with 1.72 g of TGA by magnetically stirring for 2 h at 80°C. Subsequently, the mixture was poured into 300 ml of 95% ethanol, and the TA was collected from the precipitate. TA was then lyophilized and stored at 4°C for later use.

### 5.3 The preparation of AF and TAF

For the mucus adhesion test, alginate and TA were labeled with FITC to observe under a fluorescent microscope. A 0.12 g of sodium alginate was dissolved in 12 ml of sodium acetate buffer (pH 4.9) and then mixed with 50 mg of EDC and 30 mg of NHS by magnetic stir for 30 min. A 60 mg of 1, 6‐diaminohexane was added and reacted for further 4 h to produce alginate‐amine. The alginate‐amine was collected from the precipitate and washed with 95% ethanol three times. The prepared alginate‐amine was dissolved in 30 ml of sodium bicarbonate solution (pH 9.0) and then added in 1 mg of FITC to complete the reaction in 4 h. The AF was precipitated from 100 ml of 95% ethanol.^27^ It was lyophilized and stored at 4°C for later use.

To prepare TAF, 0.12 g synthesized alginate‐amine was dissolved in 30 ml of sodium bicarbonate solution (pH 9.0) and then added in 26 mg of TGA, where the mixture was stirred magnetically for 2 h at 80°C. Thereafter, 1 mg of FITC was added into the stirred mixture and reacted for 4 h. The TAF was obtained from the precipitate of the addition of 100 ml of 95% ethanol. The precipitate was lyophilized and stored at 4°C for later use.

### 5.4 Fourier transform infrared spectroscopy

FTIR was utilized to identify the functional groups of the synthesized TA. Measurements were carried out using attenuated total reflectance‐FTIR spotlight 200i (Perkin Elmer). The sample was mounted on the stage and analyzed by transmittance mode in the range of 500–4000 cm^−1^. The functional groups corresponding to the absorption bands were identified by software Origin.

### 5.5 NMR spectroscopy analysis

The molecular structure both of alginate and TA was identified by NMR. A 10 mg of alginate or TA was dissolved in 1 ml of D_2_O. Measurements were conducted using Bruker Avance III 500 spectrometer.

### 5.6 Biocompatibility of TA

The cell viability, cytotoxicity, and cell death rate of the specimen were evaluated by water‐soluble tetrazolium salt‐1 assay (WST‐1 assay), lactate dehydrogenase (LDH) assay, and live/dead staining,^28^, ^29^, ^30^ respectively.

The 1% alginate and 1% TA were used in all biocompatibility tests, including cell viability, cytotoxicity test, and live and dead staining. A 0.2 g specimen was dissolved in 1 ml of serum‐free MEM medium as a test solution. A 0.2 g of zinc diethyldithiocarbamate (ZDEC) and Al_2_O_3_ were immersed in 1 ml of serum‐free MEM medium for 1 day where the extracted solutions were used as the positive control and negative control, respectively. L929 was first seeded onto a 96‐well plate with a density of 10^4^ cells/well. After 1 day of culture, the medium was replaced by the test solution and extracted solution and then cultured for one more day.

In the cell viability test, the medium was replaced by a medium containing WST‐1 assay, followed by another 1.5‐h culture. The culture plate was placed on an ELISA reader (SpectraMax iD3 from Molecular Devices) and evaluated the cell viability with the absorbance at 450 nm.

In the cytotoxicity test, 50 μl medium was transferred to a new 96‐well plate and added 50 μl of LDH reagent each well, followed by another 1.5‐h culture. The plate was mounted on an ELISA reader and checked the cytotoxicity of the specimen by the absorbance at the wavelength of 490 nm.

In the test of cell death rate, L929 was seeded onto a 24‐well plate with a density of 2 × 10^4^ cells/well. After being cultured for 1 day, the medium was removed and the cells were washed by PBS three times. The staining solution, Calcein AM (2 μM) and ethidium homodimer‐1 (EthD‐1) (4 μM) was added in and wrapped by a tin‐foil to keep in dark for 30 min. The plate was mounted on a fluorescent microscope at the wavelength of 488 and 525 nm for Calcein AM and ethidium homodimer‐1 (EthD‐1), respectively. The cells could be observed in green color and red color for living cells and dead cells, respectively.

### 5.7 Mucoadhesive test of TA in vitro

An in vitro model was designed to evaluate the mucoadhesive ability to stay on the small intestinal tract.^31^ First, the intestinal epithelium cells, IEC‐6, were seeded on a μ‐slide (from ibidi μ–slide I^0.8^ Luer) with 2 × 10^5^ cells per slide and then cultured for 1 day. The cell on the μ‐slides was stained in Hoechst first for 30 min and then a medium contained alginate‐FITC (AF) or thiolated alginate‐FITC (TAF) (100 mg/ml) incubated with cells for another 1 h. The μ‐Slide was then linked to a syringe pump system with a controlled medium outlet. The seeded cells were subjected to a constant flow of DMEM at a rate of 125 μl/min, which produced 0.15 dynes cm^−2^ of shear stress for 2 h. Finally, the μ–Slide was placed on the fluorescent microscope at the wavelength of 488 nm to observe how much TA adhere on the slide; 350 nm to observe how many cells would adhere on the slide after the constant flow of the medium.

### 5.8 The investigation of TA as a nutrient barrier in the small intestine

A modified Franz‐type diffusion cell was used as in vitro model to study whether the synthetic material could be the barrier to inhibit nutrient absorption.^32^ The whole design was schemed as in Figure S2. To mimic the mucus layer of the small intestinal tract, a cellulose membrane with a pore size of 6 μm (Advantec) was immersed in 3% mucin (Sigma‐Aldrich) solution at room temperature for 1 h. The membrane was taken out from mucin solution, as the mucin‐coated membrane. The mucin‐coated membrane was further steeped with PBS, 1% alginate, or 1% TA by a drip‐on process as the control group, alginate group, and TA group. The membrane was mounted into the Franz‐type diffusion cell. The donor chamber (upper part) and receptor chamber (ground part) of the Franz‐type diffusion cell would fill up 20 ml of 500 mg/dl glucose solution and 30 ml PSB, respectively. The glucose would freely diffuse from the donor chamber to the receptor chamber. After 10‐min diffusion, the glucose solution was collected and analyzed by a glucometer (Accu‐Chek Instant, Roche).

### 5.9 The mucoadhesive test of TA in vivo

C57BL/6 male mice (8‐week‐old) were gavaged with AF or TAF by 100 mg/kg to test how much adhered to the mucus layer of the GI tract. After gavage feeding for 0, 10, 30, 60, 120, and 240 min, the mice were sacrificed and the GI tract was harvested (*n* = 3 at each time point). The harvested GI tract was placed in a chamber of IVIS Luminar II (Perkin Elmer)^33^ and then checked the fluorescent intensity of Ex. 500 nm and Em. 540 nm to detect how much AF or TAF adhered to the GI tract to examine the adhesive behavior.

### 5.10 The evaluation of absorption barrier in OGTT

OGTT was a convenient and simple way to evaluate the function of the nutrient absorption barrier of TA by postprandial glucose absorption. In short, C57BL/6 male mice (8‐week‐old) were fasting for 18 h and then gavaged 100 mg/kg of TA. After 1 h, the mice were oral administration with 4 g/kg of as‐received glucose solution. The blood glucose level of the mouse was measured by glucose meter (Accu‐Chek Instant, Roche) every 30 min, until 120 min.

Mice were gavaged with PBS first, then administered with glucose solution 1 h later as the control; mice gavaged with TA first, followed by glucose solution administration 1 h later were the TA group; mice first treated with PBS first, followed by PBS administration 1 h later were the sham group.

### 5.11 The evaluation of barrier property in IPGTT

The IPGTT was to further confirm that gavaged TA to block glucose absorption was through the GI pathway rather than the other pathways. Briefly, C57BL/6 male mice (8‐week‐old) fasted for 18 h before being gavaged with 100 mg/kg of TA. After 1 h, mice were intraperitoneally injected with 4 g/kg of as‐received glucose solution. The blood glucose level of the mouse was measured by glucose meter (Accu‐Chek Instant, Roche) every 30 min, until 120 min. The control group was the mice gavaged with PBS. After 1 h, the mice were injected with glucose solution intraperitoneally; the TA group was gavaged with TA. After 1 h, the mice received glucose injection intraperitoneally; and the sham group was gavaged with PBS. After 1 h, the mice were injected with PBS into the peritoneal cavity.

### 5.12 In vivo study

The 4‐week‐old C57BL/6 male mice (BioLASCO) were acclimated for 1 week after being delivered to the animal research center. All mice were randomly separated into each cage until the end of the experiment at 10 weeks. The experiment was divided into four groups: control group, HFD group, HFD‐Alg group, and HFD‐TA group. In the control group, mice were daily gavaged with PBS and a normal diet (rodent chow) provided ad libitum. In the HFD group, mice were daily gavaged with PBS and fed with a 60% high‐fat diet (Dyets, DYET# 112252). In the HFD‐Alg group, the mice were daily gavaged with alginate 100 mg/kg and fed with a 60% high‐fat diet. In the HFD‐TA group, the mice were daily gavaged with TA 100 mg/kg and fed with a 60% high‐fat diet. Body weight and food intake were recorded every week.

At the end of the experiment, the whole‐body micro computerized tomography (micro‐CT, SKYSCAN) was used to evaluate body‐fat distribution by resolution of 35 μm.^34^

The mice were sacrificed at 10 weeks. The subcutaneous and epididymis were collected, and the white adipose tissue was harvested and isolated. The overall weight of white adipose tissue was measured and recorded. The GI tract‐related organs and tissues were harvested for histological sectioning and observed under an optical microscope by hematoxylin and eosin staining (H&E stain).

### 5.13 Statistics

The results obtained are expressed by “mean ± standard deviation.” One‐way ANOVA with multiple comparison tests was adopted for the statistical difference of the experiments. The difference was considered significant at the *p* value less than 0.05 (*<0.05; **p < 0.01; ***p < 0.001).

## AUTHOR CONTRIBUTIONS

**Tzu‐Chien Chen:** Conceptualization (equal); formal analysis (lead); investigation (lead); methodology (lead); validation (lead); visualization (lead); writing – original draft (equal); writing – review and editing (equal). **Rui‐Chian Tang:** Investigation (supporting); methodology (supporting); writing – original draft (supporting); writing – review and editing (supporting). **Jhih‐Ni Lin:** Investigation (supporting); methodology (supporting). **Wei‐Ting Kuo:** Methodology (supporting). **I‐Hsuan Yang:** Methodology (supporting). **Ya‐Jyun Liang:** Methodology (supporting). **Feng‐Huei Lin:** Conceptualization (equal); methodology (equal); supervision (lead); writing – original draft (equal); writing – review and editing (equal).

6

### PEER REVIEW

The peer review history for this article is available at https://publons.com/publon/10.1002/btm2.10382.

## Supporting information


**Figure S1** The EDS analysis for alginate and TA
**Figure S2**. The picture of modified Franz‐type diffusion cell
**Figure S3**. IVIS imaging of the mouse GI tract after TAF gavaged for 24 hours
**Figure S4**. Cell viability of alginate and TA in IEC‐6 cell line
**Figure S5**. Cytotoxicity of alginate and TA in IEC‐6 cell line
**Figure S6** Live/dead staining of alginate and TA in IEC‐6 cell line
**Figure S7**. Food intake in HFD and HFD‐TA
**Figure S8**. H&E staining of the stomach, small intestine, pancreas, and spleen

## Data Availability

The study was supported by the National Health Research Institutes (BN‐111‐PP‐01 and BN‐111‐GP‐07) and subsidized by Ministry of Science and Technology and National Taiwan University (NTU),Taiwan.

## References

[1] World Health Organization . Overweight and Obesity. WHO; 2020.

[2] Re RN . Obesity‐related hypertension. Ochsner J. 2009;9(3):133‐136. 21603428 PMC3096270

[3] Singh RK , Kumar P , Mahalingam K . Molecular genetics of human obesity: a comprehensive review. C R Biol. 2017;340(2):87‐108. 28089486 10.1016/j.crvi.2016.11.007

[4] Malik VS , Popkin BM , Bray GA , Després J‐P , Hu FB . Sugar‐sweetened beverages, obesity, type 2 diabetes mellitus, and cardiovascular disease risk. Circulation. 2010;121(11):1356‐1364. 20308626 10.1161/CIRCULATIONAHA.109.876185 PMC2862465

[5] UK NCGC . Obesity: Identification, Assessment and Management of Overweight and Obesity in Children, Young People and Adults. National Institute for Health and Care Excellence; 2014. 25535639

[6] Friedrich M . Global obesity epidemic worsening. JAMA. 2017;318(7):603. 10.1001/jama.2017.10693 28810033

[7] Uranga RM , Keller JN . The complex interactions between obesity, metabolism and the brain. Front Neurosci. 2019;13:513. 31178685 10.3389/fnins.2019.00513 PMC6542999

[8] Gloy VL , Briel M , Bhatt DL , et al. Bariatric surgery versus non‐surgical treatment for obesity: a systematic review and meta‐analysis of randomised controlled trials. BMJ. 2013;347:f5934. 24149519 10.1136/bmj.f5934 PMC3806364

[9] Goyal H , Kopel J , Perisetti A , et al. Endobariatric procedures for obesity: clinical indications and available options. Ther Adv Gastrointest Endosc. 2021;14:2631774520984627. 33629061 10.1177/2631774520984627 PMC7841245

[10] Glaysher MA , Mohanaruban A , Prechtl CG , et al. A randomised controlled trial of a duodenal‐jejunal bypass sleeve device (EndoBarrier) compared with standard medical therapy for the management of obese subjects with type 2 diabetes mellitus. BMJ Open. 2017;7(11):e018598. 10.1136/bmjopen-2017-018598 PMC5695522 29146657

[11] Storm AC , Dayyeh BKA , Topazian M . Endobariatrics: a primer. Clin Gastroenterol Hepatol. 2018;16(11):1701‐1704. 29551737 10.1016/j.cgh.2018.03.009

[12] Ruban A , Ashrafian H , Teare JP . The EndoBarrier: duodenal‐jejunal bypass liner for diabetes and weight loss. Gastroenterol Res Pract. 2018;2018:1‐9. 10.1155/2018/7823182 PMC6083488 30147720

[13] Kumar Giri T , Thakur D , Alexander A , Badwaik H , Krishna TD . Alginate based hydrogel as a potential biopolymeric carrier for drug delivery and cell delivery systems: present status and applications. Curr Drug Deliv. 2012;9(6):539‐555. 22998675 10.2174/156720112803529800

[14] Chater PI , Wilcox MD , Houghton D , Pearson JP . The role of seaweed bioactives in the control of digestion: implications for obesity treatments. Food Funct. 2015;6(11):3420‐3427. 26416783 10.1039/c5fo00293a

[15] Lange KW , Hauser J , Nakamura Y , Kanaya S . Dietary seaweeds and obesity. Food Sci Human Wellness. 2015;4(3):87‐96.

[16] Georg Jensen M , Kristensen M , Astrup A . Effect of alginate supplementation on weight loss in obese subjects completing a 12‐wk energy‐restricted diet: a randomized controlled trial. Am J Clin Nutr. 2012;96(1):5‐13. 22648709 10.3945/ajcn.111.025312

[17] Tawiah A , Cornick S , Moreau F , et al. High MUC2 mucin expression and misfolding induce cellular stress, reactive oxygen production, and apoptosis in goblet cells. Am J Pathol. 2018;188(6):1354‐1373. 29545196 10.1016/j.ajpath.2018.02.007

[18] Circu ML , Aw TY . Redox biology of the intestine. Free Radic Res. 2011;45(11–12):1245‐1266. 21831010 10.3109/10715762.2011.611509 PMC3210416

[19] Puri V , Sharma A , Kumar P , Singh I . Thiolation of biopolymers for developing drug delivery systems with enhanced mechanical and mucoadhesive properties: a review. Polymers. 2020;12(8):1803. 32796741 10.3390/polym12081803 PMC7464630

[20] Keita AV , Söderholm JD . The intestinal barrier and its regulation by neuroimmune factors. Neurogastroenterol Moti. 2010;22(7):718‐733. 10.1111/j.1365-2982.2010.01498.x 20377785

[21] Cornick S , Tawiah A , Chadee K . Roles and regulation of the mucus barrier in the gut. Tissue Barriers. 2015;3(1–2):e982426. 25838985 10.4161/21688370.2014.982426 PMC4372027

[22] Cornick S . SNARE‐Mediated Exocytosis of MUC2 from Intestinal Goblet Cells (Unpublished doctoral thesis). University of Calgary; 2018.

[23] Bettermann EL , Hartman TJ , Easley KA , et al. Higher Mediterranean diet quality scores and lower body mass index are associated with a less‐oxidized plasma glutathione and cysteine redox status in adults. J Nutr. 2018;148(2):245‐253. 29490099 10.1093/jn/nxx045 PMC6251672

[24] Nkabyo YS , Gu LH , Jones DP , Ziegler TR . Thiol/disulfide redox status is oxidized in plasma and small intestinal and colonic mucosa of rats with inadequate sulfur amino acid intake. J Nutr. 2006;136(5):1242‐1248. 16614411 10.1093/jn/136.5.1242

[25] Kothale D , Verma U , Dewangan N , Jana P , Jain A , Jain D . Alginate as promising natural polymer for pharmaceutical, food, and biomedical applications. Curr Drug Deliv. 2020;17(9):755‐775. 32778024 10.2174/1567201817666200810110226

[26] Gheorghita Puscaselu R , Lobiuc A , Dimian M , Covasa M . Alginate: from food industry to biomedical applications and management of metabolic disorders. Polymers. 2020;12(10):2417. 33092194 10.3390/polym12102417 PMC7589871

[27] Liu J , Zhang Y , Yang T , et al. Synthesis, characterization, and application of composite alginate microspheres with magnetic and fluorescent functionalities. J Appl Polym Sci. 2009;113(6):4042‐4051.

[28] Chen W‐Y , Lin F‐H . Oxidized hyaluronic acid hydrogels as a carrier for constant‐release clenbuterol against high‐fat diet‐induced obesity in mice. Front Endocrinol. 2021;12:102. 10.3389/fendo.2021.572690 PMC7996091 33776904

[29] Chi C‐Y , Chen C‐Y , Huang J‐Y , et al. Preparation and in‐vitro evaluation of Fe2O3‐doped DP‐bioglass in combination with 3D‐printing and selective laser sintering process (3DP‐SLS) for alveolar bone augmentation. Ceram Int. 2021;47(9):12725‐12734.

[30] Liang Y‐J , Hong J‐Y , Yang I , et al. To synthesize hydroxyapatite by modified low temperature method loaded with bletilla striata polysaccharide as antioxidant for the prevention of sarcopenia by intramuscular administration. Antioxidants. 2021;10(3):488. 33804703 10.3390/antiox10030488 PMC8035982

[31] Tang R‐C , Chen T‐C , Lin F‐H . Design strategy for a hydroxide‐triggered pH‐responsive hydrogel as a mucoadhesive barrier to prevent metabolism disorders. ACS Appl Mater Interfaces. 2021;13(49):58340‐58351. 34871495 10.1021/acsami.1c17706 PMC8802295

[32] Chen T‐C , Ho Y‐Y , Tang R‐C , et al. Thiolated chitosan as an intestinal absorption carrier with hesperidin encapsulation for obesity treatment. Nutrients. 2021;13(12):4405. 34959957 10.3390/nu13124405 PMC8706427

[33] Lee Y , Deelman TE , Chen K , Lin DS , Tavakkoli A , Karp JM . Therapeutic luminal coating of the intestine. Nat Mater. 2018;17(9):834‐842. 29891893 10.1038/s41563-018-0106-5

[34] Kopec AK , Abrahams SR , Thornton S , et al. Thrombin promotes diet‐induced obesity through fibrin‐driven inflammation. J Clin Invest. 2017;127(8):3152‐3166. 28737512 10.1172/JCI92744 PMC5531415

