# Supplementary material for: The synthesis and evaluation of thiolated alginate as the barrier to block nutrient absorption on small intestine for body‐weight control
Source: Bioeng Transl Med. 2022 Aug 12;8(5):e10382. doi: 10.1002/btm2.10382 (PMC10487312; doi:10.1002/btm2.10382)
Supplement: Supplementary file 1 — Figure S1 The EDS analysis for alginate and TA Figure S2. The picture of modified Franz‐type diffusion cell Figure S3. IVIS imaging of the mouse GI tract after TAF gavaged for 24 hours Figure S4. Cell viability of alginate and TA in IEC‐6 cell line Figure S5. Cytotoxicity of alginate and TA in IEC‐6 cell line Figure S6 Live/dead staining of alginate and TA in IEC‐6 cell line Figure S7. Food intake in HFD and HFD‐TA Figure S8. H&E staining of the stomach, small intestine, pancreas, and spleen [file BTM2-8-e10382-s001.docx]

Supporting Information

**The Synthesis and Evaluation of Thiolated Alginate as the Barrier to Block Nutrient Absorption on Small Intestine for Body-weight Control**


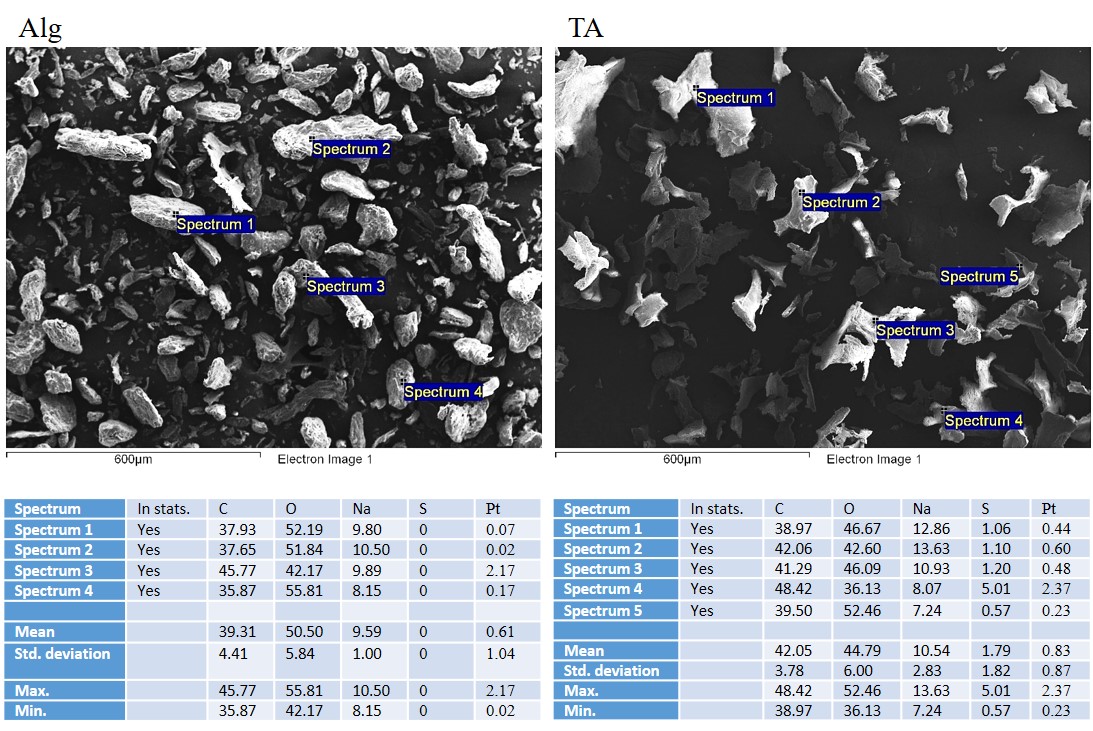


**Figure S1. The EDS analysis for alginate and TA**


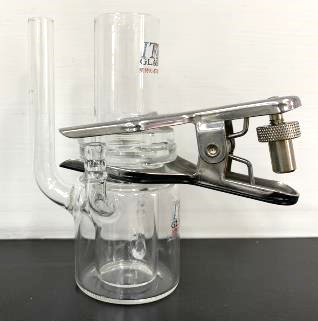


**Figure S2. The picture of modified Franz-type diffusion cell**


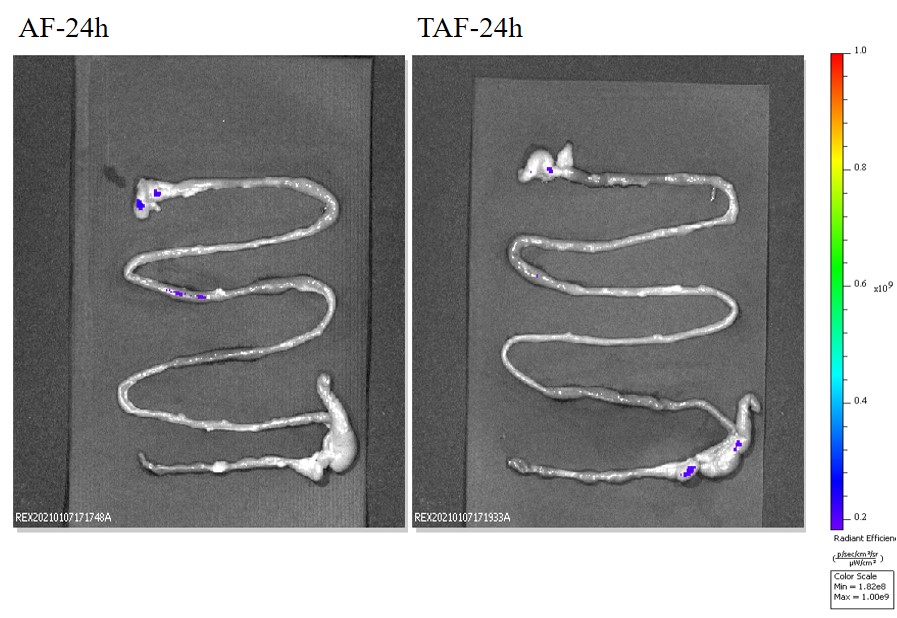


**Figure S3. IVIS imaging of the mouse GI tract after TAF gavaged for 24 hours**

^
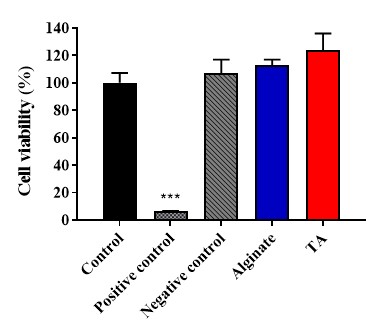
^

**Figure S4. Cell viability of alginate and TA in IEC-6 cell line**

**
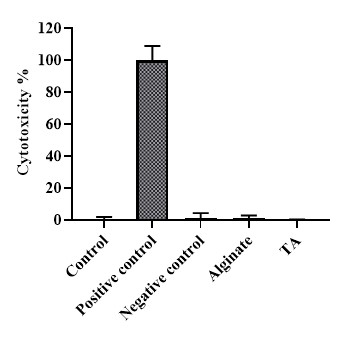
**

**Figure S5. Cytotoxicity of alginate and TA in IEC-6 cell line**


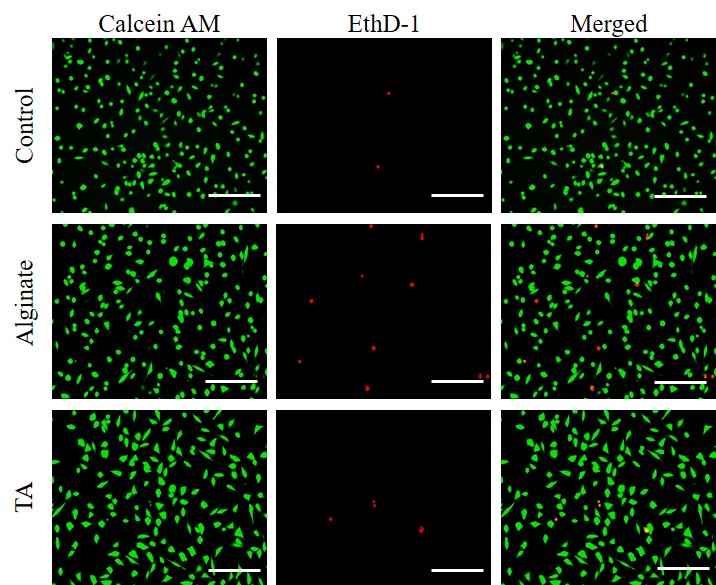


**Figure S6 Live/dead staining of alginate and TA in IEC-6 cell line**


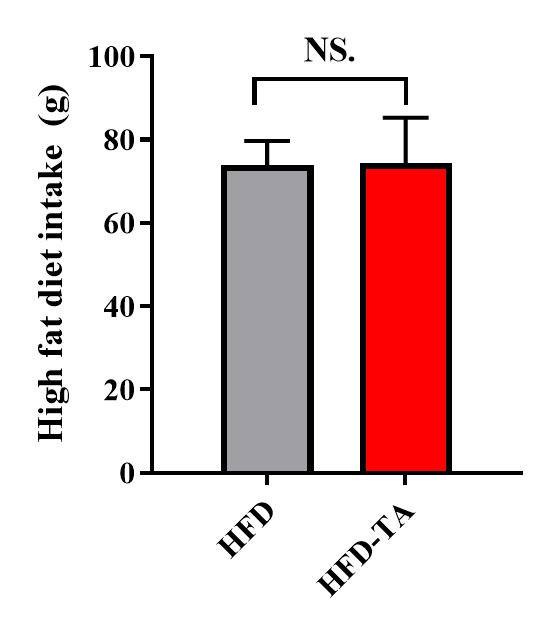


**Figure S7. Food intake in HFD and HFD-TA**


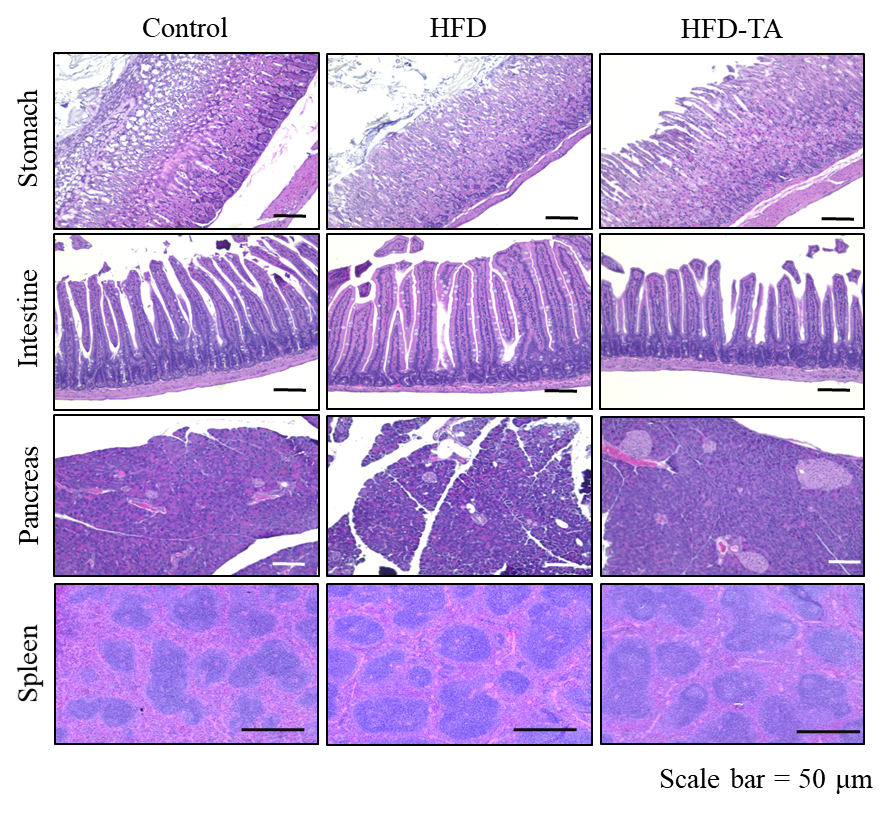


**Figure S8. H&E staining of the stomach, small intestine, pancreas, and spleen**
